# A general theory of transition to addiction it was and a general theory of transition to addiction it is: Reply to the commentaries of Ahmed, Badiani, George & Koob, Kalivas & Gipson, and Tiffany

**DOI:** 10.1007/s00213-014-3628-9

**Published:** 2014-06-08

**Authors:** Pier Vincenzo Piazza, Véronique Deroche-Gamonet

**Affiliations:** 1 Neurocentre Magendie, Physiopathologie de la Plasticité Neuronale, INSERM, U862, 146 rue Léo Saignat, 33077 Bordeaux, France; 2 Neurocentre Magendie, Physiopathologie de la Plasticité Neuronale, University of Bordeaux, U862, 146 rue Léo Saignat, 33076 Bordeaux, France

Before replying to the comments to our “multi-step general theory of transition to addiction,” we would like to thank the authors for the time and effort they have devoted to analyze our manuscript. These colleagues have raised some reasonable concerns about our theory that will be addressed in a future revised version, but we are pleased that the basic principles of our theory continue to stand.

Our reply is divided in three sections. The first section contains a discussion that addresses concerns that appeared in several commentaries. The second and third sections contain our reply to the criticisms that we agree or disagree with, respectively.

A. Common criticisms; we rediscovered the wheel and it doesn’t even works properly
  Often, when one publishes something potentially innovative one hears two basic criticisms:
  1. It is not true.
  2. It is not new, typically expressed as it was said/published before and/or everyone knows it is a known fact.
    In the case of our theory, these two criticisms are the common denominators of all of the commentaries we received. “It is not true, this is not a general theory” can be found in Badiani, Tiffany, and Ahmed’s commentaries. “It is not new” is said in Kalivas and Gipson, Ahmed, Tiffany and George and Koob’s commentaries.
    - It is not true; this is not a general theory, because it does not describe everything.
*This is normal*: *a general theory only has to provide a framework that can include everything*.
      By definition, a general theory conciliates, in a single framework, different knowledge and theories in a given field. We agree that this is an ambitious goal and when subjected to scholarly criticism, formulating a general theory is almost an impossible endeavor. However, general theories have been previously established in other scientific fields. Therefore, albeit difficult, such theories are possible. However, one issue is important to clarify. Attempting to describe all the knowledge of a field in a single paper is destined to fail and is not one of the requirements of a general theory. The utility of general theories is not to discuss and describe everything in a field; rather, they provide a frame that is able to incorporate all relevant past and future knowledge in that field. Consequently, a general theory is not judged by the pieces that have been omitted. Instead, one must evaluate whether the omitted pieces can fit within the general theory. If not, then the theory needs to be revised or dropped.
      Badiani, Tiffany, and Ahmed argue that ours is not a general theory. However, with the guidelines described above, none of their comments provide evidences supporting their claims and suggesting that our theory is false.
      Badiani makes specific and fair examples of what we have not included or exhaustively discussed. His first point is the lack of an extensive discussion of potential differences between the different drugs of abuse. This is absolutely true. However, as mentioned earlier, what would endanger our general theory is not the fact that we did not extensively describe all the drugs; rather, it is evidence demonstrating that there are drugs of abuse that cannot be incorporated in its frame. In fact, Table 2 in our paper shows that the Intensive Sustained Escalated use (ISuE) and Loss of Control (LoC) phases can be identified for alcohol, cocaine, opioids, and cannabis. Clearly, drugs are different, but it is very unlikely that there will be a drug for which there will be no individual vulnerabilities and less than three identifiable discrete steps in the transition to addiction. However, if this appears to be the case, then our theory should be restrained only to the drugs for which it is pertinent. The second point raised by Badiani is that we did not include relapse and the associated reinstatement model, which are certainly important aspects of addiction. However, we did not forget relapse and reinstatement, we purposely excluded them. Our theoretical piece, as stated in the title, concerns exclusively “transition to addiction” and does not encompass the entirety of the disease, and in particular, its chronic aspects through repeated relapse. The main reason for this choice is that we believe that there is still not enough knowledge to determine if the mechanisms responsible for transition to addiction are similar or different from the one that promote relapse. Definitely, ours is not a “general theory of addiction” but only a “general theory of the transition to it.” Finally and legitimately, Badiani mentions that we did not consider data and theories which support that addiction is not a disease, but a matter of bad choices (Heyman 2013). Clearly, this piece was omitted and should not have been, but we believe that it does not question our theory. To consider that addiction is not a disease, but solely based on bad choices would require one to defend that decision making cannot be pathological. Currently, this view, which almost considers human free will as a non-neurobiological phenomenon, is quite isolated. Many colleagues, including authors of the present commentaries [e.g., (Kalivas and Volkow 2005; Bechara 2005; Ahmed et al. 2013)…], who investigate and have shown evidence for neurobiological bases of altered decision making, will agree on this point.
      Tiffany, in his opening statement, also argues that this is not a general theory of addiction because our description “is not nearly as comprehensive as promised” and that, in particular, what is lacking is “a full consideration of the human condition of addiction.” However, Tiffany did not describe what we are missing or not considering, and how this would impair our theory. The rest of the commentary is a criticism of some concepts and implications contained in our theory, but again, there is no description of the pieces of the human condition of addiction that we are missing. In this order, Tiffany comments on: (1) The dimensional (Tiffany) versus categorial (us) vision of the different steps of drug taking; (2) The correct definition of a disease and generally, if behavioral pathologies are diseases; (3) The social implications of considering addiction as a disease; and (4) Loss of control versus decreased control on drug intake in the LoC phase. Even the concluding remark of the commentary stating “in humans, the concept of control over drug use is much more complicated, behaviorally and biologically, than the caricature model by this theory,” does not identify what is missing from our theory. In conclusion, although Tiffany raises interesting points that we will discuss in the third section of this reply, he does not provide any explicit missing pieces and does not show how they would invalidate our theory.
      In the commentary by Ahmed, we were unable to understand the precise reasons why Ahmed believes our theory is false. Initially, Ahmed seems to say (second paragraph) that it is not a general theory because it is a re-description of data. However, he opens his third paragraph by saying that this is not a fatal flaw and that the same approach has been successfully used in quantum mechanics and genetics. We are unsure how to combine these two opposing comments. In other places, Ahmed’s criticisms seem to be that we did not fully describe the methodology, we did not prove that we are right, and we did not prove that our theory is better than others. These are surprising criticisms since this is not an experimental paper in which the methodology has to be valid and the statement of the authors must be proven by results. Our paper is a “theory” based on postulates and it is meant to provide predictions. This is what should be proven right or wrong by existing and future evidence. All the evidence we provided clearly support our theory. In contrast, Ahmed does not provide any evidence that disproves it. Finally, Ahmed says that we do not capture, using his words, the behavioral “colonization” observed during addiction because our model does not contain behavioral alternatives. We do not see how this impairs our theory, since lowered sensitivity to alternative reinforcers is a DSM criterion and is clearly acknowledged in our paper. In addition, we clearly say that current models of ISuE and LoC would certainly benefit from comparisons with models including reinforcement alternatives to see if these more complex procedures provide a more complete model of human transition to addiction.
    - This general theory does not sound new: its elements have been published before.
*This is normal as it is a general theory and not a theory*; *its building blocks are previously published ideas*.
*It is not new* is certainly the major criticism in four out of the five commentaries. George and Koob discuss this at length in the “principle” section of their commentary. Kalivas says this quite clearly in his introductory paragraph. Ahmed placed it in the title and Tiffany clearly says it in his sixth paragraph. We believe that our colleagues feel this way, because a general theory cannot sound new.
      (i) *By definition the building blocks of a general theory cannot be new*
        We probably have not fully explained the differences between a *theory* and a *general theory*. A *theory* provides a new vision that attempts to explain a given phenomenon in opposition with previous or future theories. By definition, a theory must be new. In contrast, a *general theory* creates an explanatory frame by incorporating existing theories and knowledge to demonstrate that these previous findings were not a collection of opposing alternatives but rather pieces of a complex puzzle. In a general theory, the explanatory frame is new but the pieces are not. As a consequence, if a general theory does to not give a sense of “déjà vu” then it is probably not a “general theory.”
      (ii) *Is our general theory not new*?
        How does one judge if a general theory is new? As mentioned above, evaluating the novelty of its building blocks cannot do this because, by definition, they cannot be new. What has to be evaluated is the novelty of the proposed frame and its ability to accommodate knowledge of the field. More precisely, two questions should be asked: (1) Has someone already provided such a multi-step frame where specific vulnerabilities interact with specific modalities of drug intake? And (2) is there relevant knowledge in the field of addiction that cannot be accommodated in the above described frame? None of the criticisms in the five commentaries mention these critical failures of a general theory. That is, none of the commentaries identify another paper that has provided an identical all-inclusive explanatory theoretical frame of transition to addiction. And, none of the commentaries identify known aspects of the addiction process that our frame cannot accommodate. As such, we believe that the goal of our paper to provide a new “general theory” to the field of addiction still stands true.
    - Individual vulnerability to addiction is an ascertained fact.
*Sometimes facts are forgotten*.
      The comments “everyone knows that” and “it is an ascertained fact” were quite surprisingly said by George and Koob about individual vulnerability to transition to addiction. We would like to provide a few comments to this statement. First, we doubt that our colleagues can objectively deny that over the last 25 years, we have actively worked to convince the field that individual vulnerability to drug intake is an ascertained fact. Second, we doubt that they can objectively acknowledge that most of the field considers individual vulnerability to addiction.
      Here are some numbers. Currently a PubMed search with “drug addiction” as the keyword generates 227,677 entries; if “individual vulnerability” is added, it goes down to 1,619 articles. This represents 0.7 % of the total papers on addiction and is even lower if you search using “individual difference” instead of “individual vulnerability.” The mean number of articles per year using the words “individual vulnerability” in their text was close to 0 in the 1960s, 1.4 in the 1970s, and 6.9 in the 1980s. After 1989 and up to 2003, it jumps to 41 and reaches a staggering 98 per year after 2004. In the same period of time, the number of articles per year found with the keywords “drug addiction” goes from 1,000 to 2,500 in the 1960s and in the 1970s, to 8,000 after 2004. Thus, articles on drug addiction naming individual vulnerabilities reach a maximum of 1.23 % after 2004. As an ascertained fact, research on individual vulnerability in addiction has not received much attention.
      One of the possible explanations is that in previous theories of addiction [for example (Wise 1987; Jentsch and Taylor 1999; Everitt et al. 2001; Koob and Le Moal 2008a, b; Robinson and Berridge 2008)] the role of individual differences has not been emphasized enough. Our theory gives them a new, more integrated, and crucial role, and shows how essential it is to take them into account when studying addiction. Our review even contains a methodological section explaining how individual differences are an essential tool to determine if a variable is “independent” (involved in physiology) or “relevant” (a true pathophysiological factor). It is true that we did not discover individual vulnerabilities in this theoretical paper. In this paper, as we do with the rest of the current knowledge in the field, we put them in what we believe is the right perspective to highlight their crucial role.
  3. What we could have done better
    George and Koob’s and Badiani’s commentaries provide criticisms that reveal two points of our paper that need revision. Below, we discuss them separately.
    1. A better chapter on how to falsify our theory
      George and Koob and Badiani criticize the chapter where we describe how to falsify the theory. We agree that this chapter could have been written better.
      Below, we propose some possible improvements on the ideas contained in this chapter.
      - How to improve the falsifiability of the first and third prediction.
        The first prediction states that “the transition to addiction depends on an interaction between individual vulnerability and drug exposure” and “variation in the degree of these interactions cannot be seen as a fundamental fallacy of the theory” and that “one must prove that one of these two variables is not necessary to the development of pathological drug use”.
        Badiani says that the first prediction cannot be falsified because it is basically true; in our view, this is a positive feature rather than a weakness of our theory. In contrast, George and Koob say that as stated in our paper, this prediction is not falsifiable. They say that to falsify it, one should demonstrate that addiction can occur in an individual without drugs and those drugs can induce addiction without an individual. The example provided by George and Koob is quite extreme, but it clearly suggests that some rewording is needed.
        The third prediction says that “The transition to addiction is a true psychiatric disease” and that, to falsify it, one must “demonstrate that in most conditions drug, exposure is both necessary and sufficient to induce addiction.”
        Badiani states that the third prediction is basically the same thing as the first one. Indeed, both predictions put forward the idea that individual vulnerability and drug exposure are needed. The two predictions then express the same postulate, but examine it from opposite sides. George and Koob say that the use of the words “most” in the second prediction is too vague to make the prediction falsifiable. They are right, the third prediction needs rewording.
        In order to address these criticisms we then propose to unify the two predictions (first and third) and reword the criteria to falsify them in the following way:
        Prediction: “the transition to addiction is a behavioral pathology that depends on an interaction between individual vulnerability and drug exposure.”
        Falsification criteria: one must show that “in a condition of drug availability, which represents a significant portion of the ones observed in humans, the number of individuals, belonging to an outbred population, that do not develop addiction is not significantly different from the experimental error inherent in the procedure and the measurement used to assess addiction. Conversely, in the same conditions, the number of individuals that do develop addiction must be significantly superior to the one generated by the experimental error inherent in the procedure and the measurement used to assess addiction.”
        The reason that formulating the condition to disprove the prediction is complicated is because physiology and pathology are not absolute states. Physiology and pathology have developed during evolution as a selection process to a given environment. Lungs are certainly a necessary condition for life, except if we are underwater. Similarly, we do not pretend to predict what would happen under the conditions of planet Mars or in a group of individuals that were continually tethered to an operating table and administered drugs three times a day. In other words, our theory does not consider the ability to induce the transition to addiction as an inherent feature of a certain number of variables, but as an emergent feature. As such, by definition, the effects of these variables and their relationships among each other are dependent on context, as is the general theory’s validity.
      - How to improve the falsifiability of the second prediction
        The second prediction states that “transition to addiction is a process that needs at least three steps.” In other words, our theory postulates that the development of addiction is a transition between at least three different identifiable stages and that in a given population of individuals using drugs one should find individuals in one stage or the other.
        George and Koob say that this prediction cannot be falsified since a transition is passing from one state to another and unless we assume that the transition is immediate, at least two steps are necessary to perform a transition. For this reason, in their opinion, our theory cannot be falsified. We do not understand this comment. It would have been valid if we had said that there are at least two steps from stages 1 to 2. However, we said at least three, from regular drug use to excessive, and from excessive to loss of control. Thus, the theory can be falsified, if for example, someone can demonstrate that transition to addiction is only a two-step process.
        On the second prediction, Badiani says that we do not provide any clear diagnostic criteria to identify the three steps and so the theory is not falsifiable. We believe that this is not true since the identifying features of the three steps are described in page 6 of our review within the “Transition to addiction in humans is a three-step process” chapter. However, Badiani’s comment suggests that in a revised version of the theory, a more extensive description could be appropriate.
        Badiani also says that we ignored the Dopamine Dysregulation Syndrome (DDS) that produces an accelerated shift to the LoC phase. We did not cite this because it is an iatrogenic condition and as a consequence, it is not immediately pertinent to our theory. However, we should have cited and discussed DDS; we will include DDS in a revised version of the theory.
    2. We should have said that this was just a general theory and not the first one.
      Badiani says that ours is not the first general theory of transition to addiction because West and Brown (2013) already did one. We were not aware of the elegant work of West and Brown as a general theory (West and Brown 2013). We believe that it is a new theory altogether since they build their theoretical construct using the elementary components found in other theories and do not use the theories themselves. In other words, in West and Brown’s book, previous theories are more atomized than unified. In contrast, we admit that saying our general theory was the first one was a mistake resulting from unwarranted enthusiasm. In modern science nothing is really said for the first time and if one looks carefully remnants of that knowledge or opinion can be found here and there in recent or past history. As a consequence, in a revised version of the theory “first” will disappear.
C. Reply to the specific criticisms of each commentary
  1. Specific reply to Tiffany’s commentary: are we in the same dimension?
    Here we present our responses to Tiffany’s comments that have not been addressed in the previous section of our reply. Many of these comments are interesting but we do not feel that they impair or challenge the validity of our theory.
    Tiffany says that “the idea that addiction is categorically distinct from non-addictive drug use stands in contrast to the common assumption that addiction is a dimensional construct with those engaged in very heavy use only quantitatively and not qualitatively different from less experienced users (Tiffany et al. 2004; Goedeker and Tiffany 2008). Here, Tiffany raises a very interesting issue in the field of psychiatry that is the unanswered question of the dimensional versus categorial vision of psychiatric diseases. In other words, is a disease simply the extreme overexpression of otherwise normal behaviors or is it a different state from a normal condition? Tiffany clearly promotes his dimensional vision that is well respected, but also certainly not commonly accepted. It is also a straw man argument to put our theory as exclusively categorial. Quite the opposite, we believe that the frame proposed by our theory is one of the few theoretical constructs that allows for reconciliation of the dimensional/categorial debate. Thus, we postulate that in the evolution of addiction, both dimensional and categorial changes exist. There is primarily a dimensional evolution between recreational, sporadic (ReS) to ISuE phases, i.e., more dopamine and more drug intake, and principally, a categorial one between ISuE and LoC. This idea is not only based on behavioral features, i.e., a bimodal distribution in resistance to punishment that appear during the LoC phase, but also on neurobiological evidence, i.e., the inability to recover a normal LTD in animals showing addiction-like behavior. In conclusion, we do acknowledge the dimensional nature of addiction but we believe that it is most prominent during the ISuE phase but not during the entire process of transition to addiction.
    Tiffany also contests the idea that our theory demonstrates that “addiction is a true psychiatric disease.” In this paragraph, he contests the existence of psychiatric diseases in general. He says that there is no consensus of the definition of what a psychiatric disease is and that depression and anxiety disorders are not established as psychiatric diseases. Tiffany suggests Jellineck’s assertion that a “Disease is what the medical profession considers as such” as the best definition of a psychiatric disease. However, following his reasoning, one has to conclude that: (1) depression and anxiety disorders are diseases since the medical profession considers them as such, and (2) since addiction shares features with anxiety and depression, addiction is also a psychiatric disease. In conclusion, we agree with Tiffany that to date, there is not a general theoretical definition of psychiatric disease. In a revised version of the theory, we will state this clearly and add that we believe that addiction is a psychiatric disease no more and no less than depression and anxiety disorders.
    Tiffany also criticizes the idea that accepting addiction as a psychiatric disease would humanize treatment of addiction, because psychiatric diseases are stigmatizing. This is probably one of the most peculiar statements of Tiffany’s commentary. It is true that all diseases, even non-psychiatric ones, are stigmatizing to a certain degree. However, a non-disease alternative for addiction would be to view it as a vice. Vices are overtly stigmatized by our societies and the proper response to them is considered punishment and not treatment. Thus, progressing from a view in which the addict is a vicious individual that should be punished to a view in which the addict is a person with a disease that needs treatment seems to be a favorable evolution to us. In addition, in the progression of our society from sectarian to nonpartisan beliefs, we believe that diseases will be rehabilitated much more quickly than vices.
    Finally, Tiffany criticizes our theory by saying that in the final stage of addiction, there is not “loss of control” of drug intake but “less control” of drug intake. Is this a lethal blow of our theory? We do not believe so. In fact what we say is that in the final stage, the control of drug intake is low enough for drug taking to become the dominant behavior. Does the fact, put forward by Tiffany, that addicts can, in experimental conditions, decrease drug intake when given an alternative reward invalidate our vision of loss of control? We do not believe so; otherwise, we would have learned to treat addiction with chocolate or money a long time ago and solved this major problem in our society using this simple and efficient strategy.
    In conclusion, we thank Dr. Tiffany for his interesting comments; they have given us the opportunity to discuss important issues in the field of behavioral pathologies. However, none of them actually impairs the validity of our theory.
  2. Specific Reply to Kalivas and Gipson’s commentary: looking for the lost “lost opportunity.”
    We have read and re-read the elegant commentary by Kalivas and Gipson and being influenced by the title “Mourning a Lost Opportunity,” we have searched for this fatal flaw, for the opportunity we missed. However, except for the statements saying that what we describe is not really new (of which we discussed in the first section of our reply) we have not found a single criticism to any of the principles or predictions of our theory. Thus, the lost opportunity is not to be found in the fact that we did not provide a “general theory of transition to addiction.”
    Starting with their second paragraph, Kalivas and Gipson provide an interesting comment on the danger of anthropomorphism and the use of experimental models. Kalivas and Gipson have broadly interpreted the word anthropomorphism to a degree that also includes behavioral face validity models. Since we have been promoting models of transition to addiction based on face validity, maybe the lost opportunity is here; we are anthropomorphist. Anthropomorphism consists of attributing human characteristics to real or imaginary beings that are not humans. Then, it is true that behavioral face validity models are quite anthropomorphic in nature but so is practically 80 % of the research in neurobiology. The simple sentence used by Kalivas and Gipson that research in animals “allows understanding how the brain works” is, for example, extremely anthropomorphist. This phrase implies that we can understand the brain of humans by studying the brains of animals; thus, we are attributing human features to the brain of a rodent despite the fact that they are quite different in shape and size. In other words, anthropomorphist is nearly inseparable from neurobiologist.
    However, it is true that anthropomorphism should be controlled and limited. And we agree with Kalivas and Gipson that attributing human feelings to animals is clearly crossing a line. Thus, it is probably here where the lost opportunity can be found: we are attributing feelings to animals. Unfortunately, this is absolutely not the case. When we talk about mourning, need, pleasure, and desire, we do not talk about animals but about humans. We are not proposing a general theory of transition to addiction in rats or mice; our general theory is for humans in which, pleasure, desire, need, and mourning are appropriate descriptions of internal states. In addition, these terms are not derived from the behavior we see in animals but from the neurobiological changes that characterize each step of the transition to addiction. Starting from these changes, we have tried to conceptualize how these changes in brain activity could be felt by humans. In conclusion, we believe that based on current neurobiological knowledge “pleasure, desire, need, and mourning” are appropriate descriptions of the state that accompany transition to addiction in humans.
    Finally, Kalivas and Gipson criticize the fact that we have proposed the escalation model as a model of the transition from the ReS to the ISuE phase. Is the lost opportunity the one of getting rid of the escalation model? On this point, we believe that the commentary by George and Koob, a eulogy of the escalation model, is the most appropriate response to Kalivas and Gipson criticisms.
    In conclusion, Kalivas and Gipson’s commentary provided a stimulating discussion of some important issues in neurobiological research in general. However, it fell short in showing us which opportunity we lost.
  3. Specific reply to Ahmed’s commentary: a lot of heat and little light
    We read with interest the personal commentary of our neighbor and colleague, Ahmed. We have addressed above some of his comments and we of course disagree with him that our HR/LR model retarded progress in the addiction field. His comments did not provide any insight about our general theory other than to say that it is not a general theory and that results from choice-based models should be incorporated in current addiction models. As discussed above, we disagree with the former statement and as already mentioned in our paper we agree with the latter statement.
  4. Specific replay to George and Koob’s commentary
    In the quite extensive part of their comments, George and Koob do not basically criticize the fundamentals of our theory but say that we have not given enough emphasis on various aspects of the transition process.
    - Embracing hedonic state but discarding withdrawal.
      In our theory, we do not say that withdrawal should be discarded because it is irrelevant to addiction, but because we believe that studying it presents a risk. Thus, withdrawal is not a necessary or sufficient condition in the transition to addiction. Several pharmacological compounds that produce serious physical and emotional withdrawal do not induce addiction and for many drugs of abuse, withdrawal is not a clear-cut event. Consequently, we prefer to be on a safer ground and study phenomena that more closely relate to addiction. However, adding physical and/or emotional withdrawal as an additional process that contributes to the transition to addiction does not constitute a problem for our theory as it can easily embrace it as a contributor factor of excessive drug intake.
      We also believe that a number of sentences by George and Koob do not really help the cause of withdrawal. For example, “it is important to realize that the diagnostic criteria in the DSM IV or DSM V are not relevant to the understanding of the mechanisms that underlie the progression of the disease” or “the National institutes of Health no longer funds projects that rely exclusively on the DSM” and “motivational withdrawal and mourning are the same thing.” How is it possible that the criteria that allows for the identification of individuals, whose pathophysiological condition we strive to understand, can be ignored when studying such a pathological process? And we and numerous other researchers in the USA and Europe strongly disagree with the notion that NIH funding strategy reflects an ultimate scientific truth? Thirty years ago, NIDA did not fund research on the influence of individual differences, role of stress in drug use, and relapse, because they were considered irrelevant in the very strong drug-centered approach of the time. Finally, by which process can a decrease in positive motivation be assimilated to the discomfort generated by the inability to replace a behavior that has become crystallized?
    - “What happened to the brain stress systems” and a “somewhat myopic vision of the biological basis of loss of control”
      We clearly stated in our paper that it is not an exhaustive review of the neurobiological changes induced by exposure to drugs and/or influencing drug taking. The main focus is to provide a general theory of transition to addiction and not to compile the many neurobiological correlates of drug effects and drug taking. Consequently, exhaustively citing everyone was not the mission of our review. Conscious of this, we apologized in the paper to the colleagues who may feel that their work was not sufficiently cited. Clearly, this apology needs to be reworded and made clearer in a revised version of the theory and we would like to renew this apology to George and Koob that clearly resent that their work was not sufficiently cited. We did not cite their work out of disrespect, but simply because either we did not need more data to prove our point or because those data would not help to prove that point. As we have explained at length, to go from physiology to pathophysiology one has to move from the study of independent variables to the study of relevant ones. This approach is explained at length in our paper. Unfortunately, for many of the variables contained in the hundreds of papers we have not cited, it is unclear if they are independent or relevant variables. We have no doubt that this incertitude will be clarified in the future.
    - Classification of the model of escalation of drug intake as an animal model that precedes the transition to loss of control.
      We believe that this comment of George and Koob arises from a misunderstanding. As we have said, a step toward loss of control is an escalation in drug intake and maintaining a sustained level of drug consumption for a considerable amount of time. Clearly, after a while, in the population that escalates drug intake, there will be individuals that will develop loss of control. Thinking otherwise would be in contradiction with our own theory. The reason why we have proposed the escalation model as a model of the ISuE phase is because this is the way in which it is currently used, i.e., the only phenotype that the model requires to be measured is the amount of drug taken. In order to extend the model from the ISuE to the LoC phase, George and Koob should introduce in the escalation model parameters that actually measure LoC and that allows identification of the animals, among the ones that have escalated drug intake, that have developed loss of control.
      We would also like to point out that proposing the escalation model as a model of the ISuE phase we have taken a theoretical stand that is in favor of the author’s works and of the Bordeaux School of Psychobiology that has largely contributed to this model. However, this position is not shared by all leaders in the field. See, for example, the commentary by Kalivas that criticizes our theory because we have included the escalation model. It is, in fact, true that escalation is not a necessary or sufficient condition to shift to loss of control and in our own experience giving the animal’s longer access to drugs and making them escalate does not increase the final number of animals that shift into the LoC phase. However, escalation of drug intake is the very definition of going from recreational to sustained drug use. Consequently, we do not see how it can be kept out from any complete modeling of transition to addiction.
      Finally, it is kind of puzzling to be criticized by these colleagues for stating that we have produced the “best” model to date of one single step of transition to addiction (i.e., the LoC phase) while they provide a strong eulogy of their escalation model, which, in their opinion, encompasses the entire transition to addiction process.
    - Percentage of vulnerable individuals
      We believe that this very elegant part of George and Koob’s commentary is also the result of a misunderstanding. In fact, this issue was fully addressed in the online supporting materials of our 2004 Science paper. As shown in the paper, changing the cut off value did not change significantly the magnitude of the number of individuals with three positive criteria. The 20 % range is not a calculation artifact but a biological reality found in rats as well in humans.
  5. Specific reply to Badiani’s comment: DSM is not an umbrella but a trampoline.
    We have been impressed by Badiani’s scholarly comments. Nevertheless, the section “Defining of Addiction,” using Badiani’s words, is “less good than the rest.”
    Badiani starts by saying that despite our best efforts, we give a circular definition of addiction because we use the DSM criteria for dependence. More accurately, what we say is that we define addiction as the collection of behaviors that are used to identify patients suffering from drug dependence in the DSM IV and substance abuse disorders (SUD) in the DSM V. We do not believe this argument is circular. What we provide is a very simple and basic operational definition that is no more circular than saying that a reinforcer is a stimulus that increases the probability of the appearance of a behavior. Similarly, we say that addiction is a condition that increases the probability of a certain number of behaviors to appear. What could be argued is that ours is not an absolute definition of addiction but a relative, medically oriented one, derived from the patients and not from an a priori theoretical construct. This is certainly the case and we believe, as argued in our paper, that it is the best approach if the goal of the research is to understand a disease.
    Badiani then says that when we use and discuss the DSM, we enter in dangerous terminological waters. We agree on the danger but we do not see when and where our ship sunk. Thus, we are quite precise in our comparison of the DSM IV and V, taking in to account, among others, all the points raised by Badiani. Importantly, we included the fact that because of the inclusion of “craving items” in DSM V, it is theoretically possible to make a diagnosis of severe SUD in the DSM V without any of the items of dependence from DSM IV (Table 1). However, we also show in Table 2 (not cited by Badiani) that this does not happen in reality. Thus, across several drugs, diagnosis of drug abuse in the DSM IV corresponds to 99 % of cases of diagnosed mild or moderate SUD (inferior to severe) with the DSM V. Similarly, a diagnosis of drug dependence with the DSM IV corresponds across most drugs in 85–93 % of cases to a diagnosis of severe SUD with the DSM V, except for cannabis, where it is 67 %. Finally, we do not use the Research Domain Criteria (RDoC) approach as a source of diagnostic criteria but try to see what clinical endpoints putative RDoC dimensions of addiction would produce. Based on this analysis, we argue that transition to addiction is a three-step process and that the different degrees of severity of the DSM V reflect different behavioral alterations mediated by different mechanisms and vulnerabilities. We do not use the DSM as a protective umbrella, as Badiani noted, but as an analytical starting point to develop our own theoretical construct.
    Finally, there is also a mistake in Badiani’s commentary when he says that we turned the DSM V rationale on its head by suggesting that abuse and dependence are “conceptually different categories reflecting a different realm of problematic use.” We do not suggest this; rather, we discussed what the justification was in the DSM IV for proposing the two categories.
D. Conclusions: if farewell is premature then greetings
  Badiani says that the farewell at the end of our paper is premature. Our response is that if farewell at the end of a paper is surprisingly not appropriate, we hope that all the authors of these commentaries would at least accept our greetings.
